# Towards sustainable multidisciplinary renal cancer tumour boards: redesigning the referral pathway

**DOI:** 10.1111/bju.70451

**Published:** 2026-10-01

**Authors:** Bhargab Ghoshal, Sarika Nalagatla, Max Roberts, Adam Mann, David Cullen, Victoria Horsfall, Andrea Da Silva, Abhishek Reekhaye, Prasad Patki, Ravi Barod, Faiz Mumtaz, Axel Bex

**Affiliations:** ^1^ University College London Medical School London UK; ^2^ Department of Urology The Royal Free London NHS Foundation Trust London UK; ^3^ Division of Surgery and Interventional Science University College London London UK

**Keywords:** multidisciplinary team meeting, kidney cancer, renal cell carcinoma, cancer service redesign, cancer referral pathway, clinical workflow

## Abstract

**Objectives:**

To develop and evaluate a complexity‐stratified referral pathway aimed at improving the sustainability, efficiency and resource utilisation of specialist multidisciplinary team (SMDT) meetings within a high‐volume tertiary renal cancer service.

**Patients and Methods:**

We performed a retrospective cohort study of benign and malignant renal cancer cases discussed at SMDT meetings between January and December 2023 at the Royal Free Specialist Centre for Kidney Cancer. Patient demographics, tumour characteristics, SMDT discussions and management outcomes of patients were analysed to identify protocolised lower complexity cases suitable for alternative MDT pathways. Based on these findings, dedicated Standard of Care (SoC) and Benign MDTs were developed and implemented to manage lower complexity renal cases outside the main SMDT. The impact of the revised referral structure on SMDT workload, discussion efficiency and specialist resource utilisation was subsequently evaluated.

**Results:**

In 2023, 2228 patients with benign and malignant renal pathology underwent 4154 SMDT discussions. Most patients (96.3%) required under seven discussions. Patients aged >70 years with renal masses <2 cm frequently underwent repeated SMDT review, despite surveillance or conservative management representing the most common management recommendation. Bosniak I–II cysts were predominantly discharged, whilst Bosniak IIF cysts were mainly managed with surveillance. Following implementation of the revised referral structure incorporating SoC and Benign MDTs, cases discussed at the main SMDT decreased from a mean of 83 to 63 patients with cancer per week, whilst discussion time per patient increased by 30.8%. The revised pathway also reduced the requirement for full SMDT attendance for protocolised cases.

**Conclusion:**

Complexity‐stratified MDT referral structure improved efficiency and sustainability by diverting lower complexity protocolised cases to dedicated Benign and SoC MDTs. This enabled the main SMDT to prioritise clinically complex decision making and optimise utilisation of specialist multidisciplinary expertise.

**Abbreviations**
AML: angiomyolipoma
EAU: European Association of Urology
(S)MDT: (specialist) multidisciplinary team
RF SCKC: Royal Free Specialist Centre for Kidney Cancer
SoC: Standard of Care
SABR: stereotactic ablative radiotherapy
SRM: small renal mass
UTUC: upper tract urothelial carcinoma

## Introduction

Kidney cancer is the sixth most common cancer in the UK, accounting for ~4% of all new cancer diagnoses, with around 14 500 new cases diagnosed annually between 2019 and 2021 [1]. Over the last decade, advances in the management of localised and metastatic RCC have substantially expanded treatment options and increased the complexity of clinical decision‐making. The delivery of high‐quality RCC care therefore requires coordinated input from multiple specialists, including urologists, oncologists, radiologists, pathologists and allied health professionals. Specialist multidisciplinary team (SMDT) meetings have consequently become central to renal cancer management. Reported benefits include improved staging accuracy, evidence‐based treatment planning, increased clinical trial recruitment, enhanced communication between specialties and improved patient satisfaction [2].

Alongside increasing therapeutic complexity, there has been significant reorganisation of cancer services within the NHS, with progressive centralisation of specialist urological oncology care [3]. As part of major service reconfigurations across London, Royal Free Specialist Centre for Kidney Cancer (RF SCKC) was commissioned by NHS England in 2014 to centralise renal cancer services across North Central and Northeast London, serving a population of ~4 million people [4]. The RF SCKC operates via a hub and spoke referral model, incorporating 16 partner hospitals and one external Trust in Wales. All referred cases undergo SMDT review. The centre manages the full spectrum of renal pathology, including small renal masses (SRMs), complex cystic disease and upper tract urothelial carcinoma (UTUC). Although commissioned for renal malignancy, benign upper tract pathology is also referred to the SMDT to centralise upper tract surgery. Annual surgical activity at RF SCKC typically exceeds 530 renal operations per year. Whilst centralisation aims to improve quality and standardisation of care, escalating referral volume and increasing oncological complexity place considerable strain on existing MDT structures.

Increasing concern has emerged internationally regarding the sustainability of conventional MDT models. A recent multi‐society briefing endorsed by several UK professional organisations argued that the practice of discussing every patient with cancer within a full MDT framework is increasingly unsustainable, resource intensive and may contribute to delays in care delivery [5]. The report advocated prioritising MDT review for clinically complex or ambiguous cases, whilst protocolised lower complexity cases could be managed through streamlined pathways supported by standardised guidance and triage systems [6]. Furthermore, prolonged MDT meetings have been associated with cognitive fatigue, logistical inefficiencies and declining quality of decision making as meetings progress [7]. Within renal cancer services, a substantial proportion of cases follow predictable or protocolised management pathways, including Bosniak IIF cyst surveillance, active surveillance of SRMs and benign upper tract pathology. However, these cases continue to consume significant specialist MDT resources despite limited requirement for complex multidisciplinary deliberation.

This study evaluates the impact of a complexity‐stratified MDT referral structure developed within a high‐volume tertiary renal cancer centre that also manages benign cases. We aimed to identify cohorts of lower complexity renal cases suitable for protocolised specialist review outside the primary SMDT and assess the impact of this revised pathway on workload, discussion efficiency and specialist resource utilisation.

## Patients and Methods

To identify number and proportions of lower complexity renal cases, we conducted a retrospective analysis of benign and renal cancer cases discussed at the RF SCKS SMDT meetings between January 2023 and December 2023. Subsequently, we measured SMDT volumes at baseline and after implementation of the revised referral pathway from October 2024 to November 2025 to assess its impact. Cases were included if complete MDT documentation was available. SMDT meetings were chaired by a consultant urologist serving as the lead SMDT chair and included consultant urologists, medical oncologists, radiologists, clinical nurse specialists and urology fellows. The meeting took place once weekly from 9:00 AM to 12:30 PM.

### Data Extraction and Quality Assurance

The SMDT data were extracted from the institutional MDT referral and data capture platform VitalData (VitalPulse Ltd, Chelmsford, UK). Additional or missing information was obtained from the electronic patient record (EPR) system Cerner Millennium (Oracle Health, Austin, TX, USA). Clinical and MDT data were also extracted from the Somerset Cancer Register (SCR version 24.1; Somerset NHS Foundation Trust, Taunton, UK), an NHS‐developed electronic cancer management platform used to document and generate SMDT recommendations. Quality assurance was undertaken through review of unclear or incomplete data entries within VitalData, with independent data validation performed by four reviewers (B.G., M.R., A.M., and S.N.).

### Study Variables

Patient demographics included gender, height, weight, Charlson Comorbidity Index, American Society of Anesthesiologists grade, metastatic status, prior chemotherapy or radiotherapy, and previous abdominal surgery. Tumour characteristic included renal mass laterality, size, histology, clinical staging, and biopsy status. SMDT discussion details collected entailed patient age at discussion, tumour stage by TNM, discussion date, and the agreed SMDT recommendation. Information about the first clinic consultation included the date of initial clinic review and the management decision documented during that consultation. Postoperative data were collected, including specimen laterality, size, histological subtype, pathological staging, and date of postoperative consultation.

### Study Definitions

A ‘new referral’ was defined as a patient first discussed in 2023. Age was defined as age at first SMDT discussion. ‘Definitive management’ referred to the overarching management strategy recommended across all SMDT discussions. Where available, histology from surgical specimens was used in preference to biopsy findings. Renal cysts were classified according to the Bosniak 2019 system. Figures were generated and statistical analyses were performed using R version 4.3.2 (R Foundation for Statistical Computing, Vienna, Austria), using relevant packages.

### Focused Analyses of Cases

#### All Cases

For all cases discussed at the SMDT, several epidemiological and quality indicator metrics were evaluated, including:

- Proportion of cases in which a definitive management decision was reached
- Number of SMDT discussions per patient
- Reasons for repeated or prolonged case discussions
- Number of cases with incomplete TNM staging

#### Low‐Complexity Cases

A key objective was to evaluate the case types that may not require the full expertise of a SMDT. These included T1a renal tumours, Bosniak renal cysts and benign renal pathology. Based on benign characteristics, additional analysis included:

- Frequency of asymptomatic Bosniak I–II cysts discussed at the SMDT
- Number of patients aged >70 years with renal tumours <2 cm discussed at the SMDT

### Revised Referral Structure

We introduced a revised MDT referral structure for renal cancer cases at the RF SCKS, whereby not all cases were routinely discussed at the main SMDT. Instead, two additional revised MDT forums were established to manage lower complex cases: the Benign MDT and the Standard of Care (SoC) MDT. These meetings were held sequentially on a weekly basis, with a combined duration of ~2 h. To reduce resource utilisation, each meeting was undertaken by a focused team consisting of a consultant radiologist, consultant upper tract urological surgeon, renal cancer clinical nurse specialist and MDT coordinator, rather than the full specialist MDT. For flexibility, both MDTs had the option of on‐line attendance. Inclusion criteria for the revised referral structure are shown in Table 1. The Benign MDT accepted referrals for benign renal tumours such as angiomyolipoma (AML) and oncocytomas, as well as other non‐cancerous renal conditions. The SoC MDT reviewed cases that were deemed at low risk of progression and would be amenable to standardised recommendations; complex T1a cases (e.g., due to solitary kidneys, bilateral tumours, previous renal intervention [such as partial nephrectomy or ablative treatment], imperative nephron‐sparing indication, or complex tumour anatomy) were discussed in the main SMDT instead of the SoC MDT.

**Table 1:** Inclusion criteria for the additional MDT forums.

MDT inclusion criteria	
Benign MDT	SoC MDT
Benign renal tumour subtypes including AMLs and oncocytomas	Incidental cyst findings from other specialties, which require an expert opinion as to the nature of a renal cyst
Benign upper tract conditions (e.g., PUJ obstruction)	Bosniak IIF cysts
Non‐functioning kidneys, considered for benign nephrectomy	Indeterminate renal lesions <2 cm
	Uncomplicated T1a renal lesions, including Bosniak III and IV cysts ≤4 cm
	Review requested by a member of the kidney cancer team to ascertain queries (e.g., growth of lesion or re‐classification of cyst)

Referrals were required to include appropriate cross‐sectional imaging (kidney protocol CT or renal MRI) alongside renal function blood tests. In AML cases, a confirmed diagnosis, either histological or radiological (CT or MRI), was required prior to MDT discussion. This approach was implemented to minimise delays and avoid cases being deferred due to incomplete clinical information.

The revised MDT referral structure was initially introduced as an 8‐week pilot in December 2024 to evaluate the impact of separating lower complexity cases from the main SMDT. Following the successful pilot phase, the pathway was formally implemented in January 2025. Any case with diagnostic uncertainty, management ambiguity or clinician concern could be escalated for discussion at main SMDT, ensuring ongoing specialist oversight and maintenance of patient safety.

### Standardised Recommendations

For T1a renal lesions discussed in the SoC MDT, all standard management options were considered appropriate, with final decision‐making individualised at clinic review. These options included renal mass biopsy, active surveillance, partial nephrectomy, percutaneous cryotherapy, or watchful waiting depending on tumour and patient factors.

Bosniak IIF cysts were recommended for interval imaging with a CT scan at 6 months, followed by annual CT surveillance for a total duration of 5 years. Management of Bosniak III and IV cysts was guided by the European Association of Urology (EAU) renal cancer guidelines [8]. Management options for Bosniak III and IV cysts measuring <4 cm followed recommendations for T1a renal masses. However, renal mass biopsy was not routinely recommended for Bosniak III lesions. Therefore, management options were generally limited to active surveillance or partial nephrectomy.

Solitary AMLs measuring <3 cm without high‐risk vascular features were recommended for discharge, provided there were no additional features necessitating surveillance. Intervention, either selective arterial embolisation or nephron‐sparing surgery, was recommended in accordance with EAU guidance [8] for larger lesions, symptomatic patients (e.g., persistent pain or bleeding), women of childbearing age, or where access to emergency care or follow‐up may be limited. Indeterminate renal lesions were recommended for interval surveillance imaging at 3–12 months depending on size to assess for interval growth and evolution in radiological characteristics.

## Results

### Clinical and Pathological Characteristics of Cases

In 2023, prior to the implementation of SoC and Benign MDT, there were a total of 4154 discussions (Table 2), with 92% of discussions involving patients with a suspected malignancy. New referrals accounted for 40.5% (*n* = 1541) of discussions about patients with cancer. The remainder of discussions related to patients who had been reviewed previously either within the same year or earlier. Overall, in 2023, there was an average of 80 discussions per week in the SMDT meetings.

**Table 2:** Characterisation of discussions in renal cancer SMDT meetings in 2023 at the RF SCKC.

	Patients, *n* (%)			Discussions, *n* (%)		
	With cancer	Without cancer	Total patients	Cancer discussions	Non‐cancer discussions	Total discussions
New referrals	1541 (75.1)	169 (96)	1710 (76.8)	1541 (40.5)	169 (48.8)	1710 (41.2)
Re‐discussions	511 (24.9)	7 (4)	518 (23.3)	2267 (59.5)	177 (51.2)	2444 (58.8)
Total	2052	176	2228	3808	346	4154

Across 2023, a total of 2228 patients were discussed in the renal cancer SMDT meetings (Table 2). In all, 92.1% (*n* = 2052) of patients had a suspected malignancy, with 75.1% of these patients as new referrals made that year, and 6.2% (*n* = 139) of patients had metastatic disease. The mean (range) age of patients reviewed during the SMDT meetings was 65.7 (17–101) years.

Histological data were obtained from either renal mass biopsies or postoperative surgical specimens. Across the cohort, the most common malignant subtypes encountered were clear cell RCC (41.2% of all histological sub‐types), papillary RCC (8.0%), and chromophobe RCC (4.7%). The most common benign subtypes included oncocytoma (6.0%), AML (20.3%), and metanephric adenoma (0.19%). In all, 4.7% of tumours were classified as ‘other’ histology, and a further 1.1% were determined to have inconclusive histology. A total of 164 UTUC cases were also discussed in 2023 within the renal cancer SMDT meetings, and UTUCs comprised 13.8% of tumours, which were biopsied during an ureterorenoscopy.

Clinical staging of renal masses was determined following radiological review. The most frequently encountered stage was T1a (40.6%), followed by T3a (12.0%). Renal cysts accounted for 589 SMDT discussions, of which only 25.1% were classified as Bosniak III or IV.

### Distribution of Discussions in SMDT Meetings

For patients discussed in 2023, 46.0% of patients required only one discussion overall, with 181 (8.1%) patients requiring at least six discussions (Fig. 1). This included both cancer and non‐cancer cases. Delving deeper into the discussions for cancer cases individually revealed that 28% (*n* = 1066) of those discussions were identified as having incomplete data. We analysed the discussions where patients required at least 10 discussions and found that 17.9% of discussions were due to incomplete imaging and a further 9.3% of discussions were due to incomplete histology data. In all, 11.1% of discussions were necessary due to insufficient time to discuss the case in the previous week. Discussion with the patient in clinic was suggested in 23.5% of discussions. Only 27.2% of discussions had an agreed upon management plan.

**Fig. 1:**
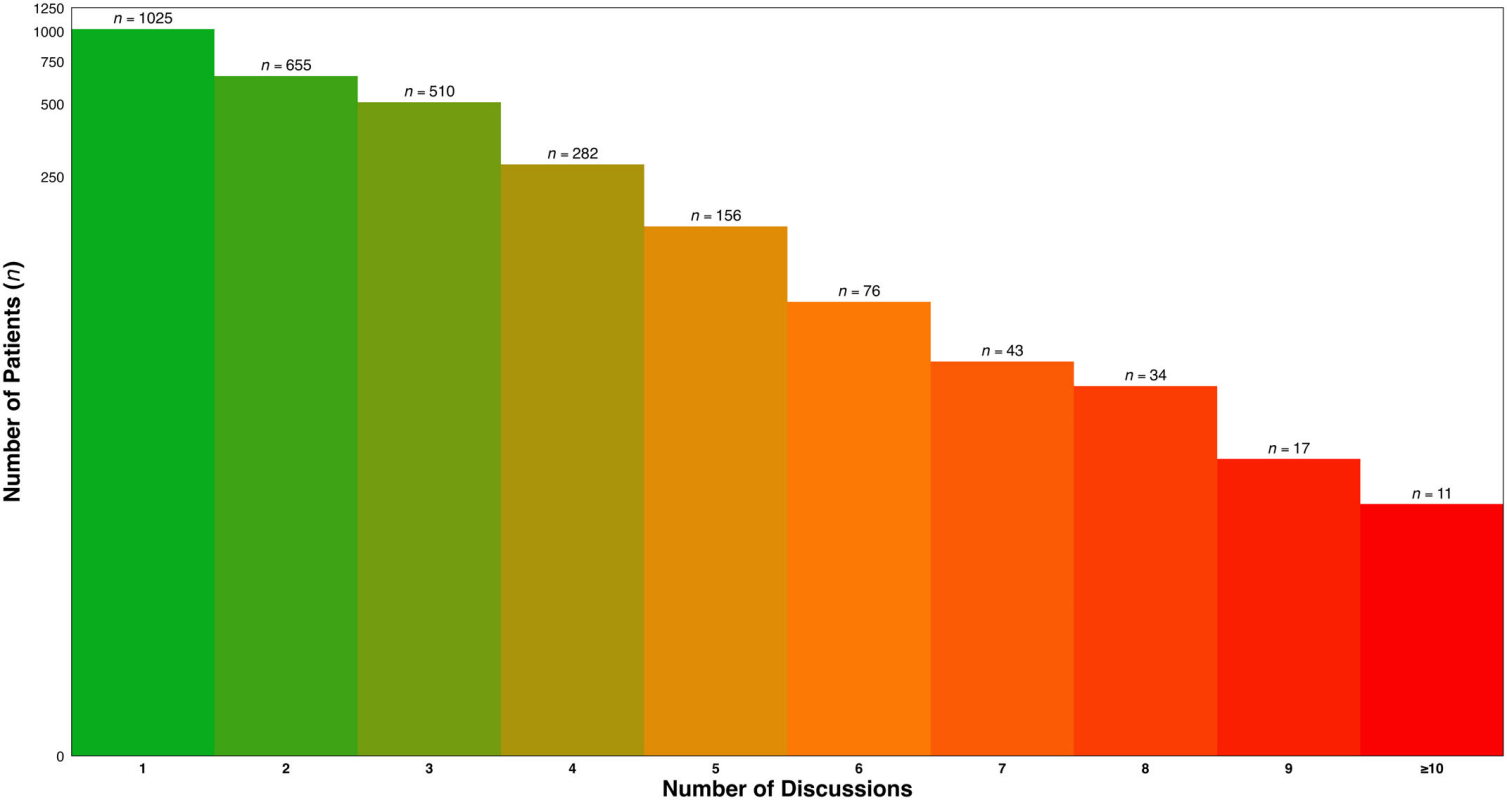
Number of discussions needed overall by patients discussed in 2023.

To further explore pattern of SMDT discussion, we evaluated a cohort of patients aged >70 years with renal masses <2 cm (the ‘elderly SRM cohort’). On analysis, 56.9% (*n* = 160) required one discussion, whilst 24.2% of patients in this cohort (*n* = 68) required at least three discussions.

We analysed the discussions where patients required more than five discussions and found that 20.2% of those discussions were due to incomplete imaging, a further 10.6% of discussions were due to incomplete histology data, and 13.8% were due to insufficient time to discuss the case in the previous week. Only 19.1% of discussions had an agreed upon management plan, with a further 16.0% of discussions re‐affirming decision to follow‐up.

We assessed the management recommendations for the elderly SRM cohort (Fig. 2). Active surveillance was the most common management strategy, accounting for 38.4% of cases (*n =* 108), whilst <19% (*n* = 51) were advised to undergo treatment, either medical or surgical, including stereotactic ablative radiotherapy (SABR) and cryotherapy. A further 38.8% (*n* = 109) of patients were discharged from the RF SCKC without any additional intervention recommended. Reasons for discharge included watchful waiting (37 patients), follow‐up no longer required (31), and care transferred back to referring centre (nine).

**Fig. 2:**
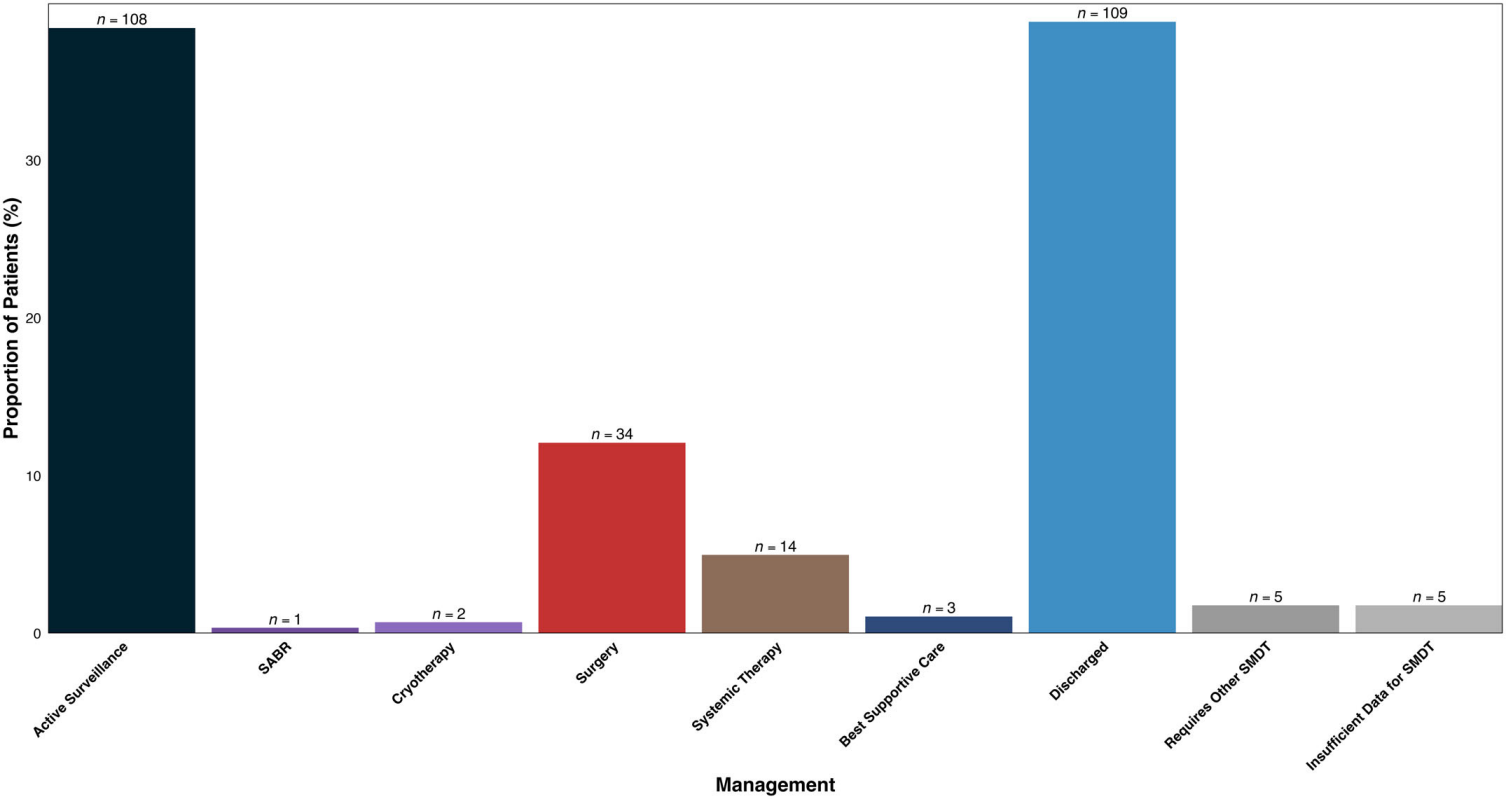
Management decisions for patients aged >70 years with tumours <2 cm.

### Management of Renal Cancer Cases

#### Management of Renal Cysts and Upper Tract Cancers

The renal cancer SMDT meetings in 2023 discussed 300 patients with Bosniak I–II cysts and 151 patients with Bosniak III or Bosniak IV cysts. Bosniak I–II cysts were typically discharged, with a minority requiring intervention for symptoms (two patients) or due to patient preference (one). Bosniak IIF cysts accounted for 119 cases and were typically put on surveillance (*n* = 94). In all, 13 patients were discharged from follow‐up, with six patients on watchful waiting. Only three patients were subsequently recommended a partial or radical nephrectomy due to changes in radiological appearance, resulting in the upstaging of classification. In contrast, surveillance was recommended for 57% and 54% of Bosniak III and IV cysts, respectively. Surgery was recommended for 25% of patients presenting with a Bosniak IV cyst, irrespective of size. Notably, 8% of patients opted for SABR or cryotherapy instead of surgery for Bosniak IV cysts.

The UTUCs were also reviewed in the SMDT meetings. The most common definitive management recommendation was nephroureterectomy, accounting for 61.2% of decisions. In contrast to renal cancers, surveillance was rarely advised for UTUC, with upfront systemic therapy offered to the 15.8% of cases presenting with metastatic disease.

#### Management of Renal Tumours

In 2023 there were 1349 renal tumours discussed and we analysed the outcomes of the SMDT meetings for these cases.

We looked closely at the difference in management options chosen for patients with T1a and T1b tumours, breaking down the management option chosen by age (Figs S1 and S2). Between the T1a and T1b cohort, there was a significant difference in management plan agreed upon. In patients with T1a tumours, active surveillance and watchful waiting was recommended more often in comparison to patients with T1b tumours, and this was more commonly seen in patients aged >70 years, with the proportion recommended for a partial nephrectomy higher in those aged <60 years compared to the rest. Conversely, in patients with T1b tumours radical nephrectomy was the most common management option across all age groups. Nonetheless, of patients that were deemed suitable for a nephron‐sparing approach, partial nephrectomy was chosen in 47.9% of patients aged <70 years, whilst in patients aged >70 years, SABR and cryotherapy was considered in 31.5% of patients.

We also broke down the management chosen for patients with renal tumours in stages T3a and above (Fig. S3). There was no substantial difference in management decision between age groups, with radical nephrectomy the mainstay management option across all age groups.

### Revised MDT Referral Structure

We reviewed the volume of cases discussed across all the MDT meetings before the full introduction of the new referral structure and the equivalent period after to analyse the impact of the new MDT referral structure.

The introduction of the new referral structure resulted in a decline in the number of cases discussed in the SMDT meeting (Fig. 3). Using the old referral structure resulted in an average of 83 patients discussed per week in the period October to December 2024 (Table 3) and in the month after the pilot period, the mean number of total cases discussed decreased to 63 per week. In comparison, the Benign MDT discussed a mean (SD) of 11 (10.2) cases whilst a mean (SD) of 8 (8.2) cases were discussed in the SoC SMDT every week in the post pilot period. On average, no more than one case each week from the Benign and SoC MDT was required to be discussed in the subsequent main SMDT.

**Fig. 3:**
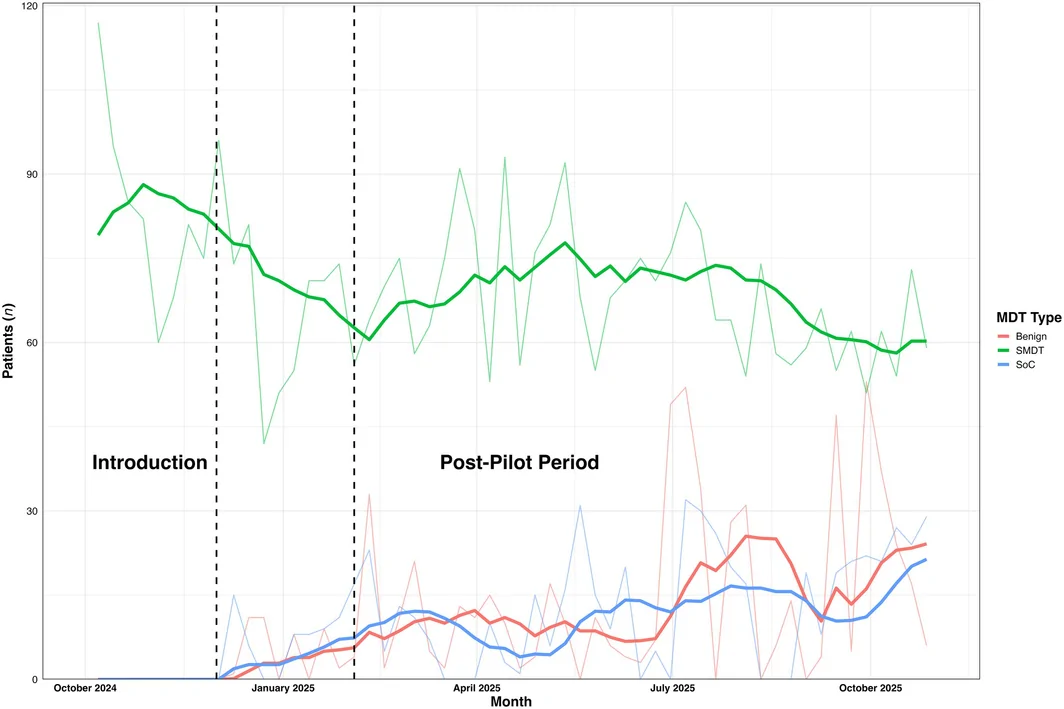
Number of patients discussed per week in the main SMDT (green), SoC MDT (blue), and Benign MDT (red) before and after the introduction of the SoC and Benign SMDTs (4‐week moving average in bold).

**Table 3:** Number of patients and time dedicated for each patient at each meeting in the main SMDT before and after the introduction of the SoC and Benign SMDTs.

SMDT meeting volume		
Period	Patients, *n*, mean (SD)	Time per patient, min, mean (SD)
October 2024 – November 2024	83 (17.4)	2.6 (0.5)
October 2025 – November 2025	63 (8.3)	3.4 (0.4)

We also reviewed how much time was dedicated per patient per month across all the MDT discussions (Table 3). The introduction of the new referral system resulted in 30.8% more time to discuss each patient in the main SMDT meeting in an equivalent period (2.6 min to 3.4 min per patient), with no change to the duration of the MDT meetings.

## Discussion

The SMDT meetings are fundamental to contemporary renal cancer care, enabling collaborative decision making for increasingly complex oncological pathways. However, the level of MDT activity highlighted in this analysis raises important questions regarding the long‐term sustainability of conventional multidisciplinary review models.

Centralisation of renal cancer services has improved access to specialist expertise and facilitated delivery of complex care across regional networks [4]. However, it has also resulted in highly heterogeneous MDT case mixes, ranging from benign renal pathology and indeterminate lesions to metastatic RCC and UTUC. Although multidisciplinary review remains essential for complex oncological decision making, our findings demonstrate that a substantial proportion of cases followed predictable management pathways with limited deviation in final recommendation despite repeated discussion.

The SMDT meetings rely on complete documentation for effective clinical decision making. However, 1929 discussions were identified as having incomplete data, including cases where histopathology or cross‐sectional imaging was unavailable at the time of discussion. Given the substantial volume of cases reviewed each week, there is limited time available for detailed pre‐meeting case preparation and screening for missing investigations or imaging. This may have contributed to the 8.1% of patients requiring six or more discussions, particularly where definitive management decisions could not be made due to incomplete information. Whilst pre‐screening meetings to ensure all MDT information is available may improve data completeness, this is unlikely to be practical within a high‐volume tertiary service. However, due to practicality and resource constraints, currently a urologist of the specialist team ensures that the minimum datasets are present.

Another notable finding was the volume of lower complexity cases discussed within the SMDT. Studies such as those by Pierorazio et al. [9] showed that active surveillance was non‐inferior to primary intervention in older patients with SRMs. However, the elderly SRM cohort were frequently reviewed multiple times despite active surveillance or watchful waiting ultimately being the predominant recommendation. Similarly, whilst Bosniak IIF cysts were predominantly managed with interval surveillance imaging, Bosniak I–II cysts were almost universally discharged. Asymptomatic Bosniak I–II cysts should not have been referred to the SMDT in the first place but were added because of ambiguous radiology reports without Bosniak classification. These observations suggest that many cases discussed within the main SMDT are suitable for protocolised specialist review pathways rather than requiring repeated full SMDT discussion.

Following implementation of the Benign and SoC MDTs, the number of patients discussed within the primary SMDT decreased, whilst discussion time per patient increased by 30.8%. This is particularly important given evidence that prolonged MDT meetings are associated with cognitive fatigue and reduced quality of decision making as meetings progress [3, 5]. Although the duration of the main SMDT remained the same, by redirecting protocolised lower complexity cases into focused specialist pathways, the main SMDT was able to dedicate greater attention to clinically challenging oncological cases. The mean discussion time per patient only increased from 2.6 to 3.4 min, but this difference should be interpreted in the context of the large number of cases reviewed, the majority of which required minimal discussion. This time saved cumulatively allowed more time to be devoted to a relatively small number of complex patients, thereby increasing the depth of multidisciplinary deliberation for cases requiring more detailed consideration. It also meant that less complex cases, which previously would not have received any MDT attention, are now briefly reviewed and formally signed off by a member of the specialist team through the Benign and SoC MDTs.

The revised pathway also improved specialist resource utilisation. The Benign and SoC MDTs operated using a focused four‐member specialist team rather than the full tertiary MDT complement, representing a more efficient use of workforce resource. This is increasingly relevant in the context of ongoing workforce shortages across cancer services [6].

Management patterns observed across tumour stages further support a stratified approach to MDT review. T1a tumours demonstrated the greatest variation in management strategy. In contrast, although higher stage tumours were more consistently managed surgically, these cases required substantially greater multidisciplinary consideration regarding operative feasibility, metastatic burden, systemic therapy integration, and patient fitness.

Our findings are consistent with previous studies demonstrating inefficiencies within universal MDT discussion models. Mullan et al. [10] showed that many early‐stage head and neck cancer cases required limited MDT deliberation, whilst Acher et al. [11] reported that MDT discussion did not alter management in a substantial proportion of urological cancer cases. Similarly, Merker et al. [12] demonstrated improved efficiency following introduction of structured referral and pre‐screening processes within breast cancer MDTs. Together, these studies support increasing adoption of selective and complexity‐based MDT pathways across oncology services. Artificial intelligence (AI) can also improve MDT efficiency, including in pre‐screening and decision‐making. Tools such as TrustedMDT, developed by Oxford University Hospitals’ Department of Oncology [13, 14], can analyse patient health records, aid radiological tumour staging, and support guideline‐based management decisions. These tools can significantly strengthen pre‐screening, ensuring referrals are appropriate and submitted with complete imaging and histology, whilst also grounding MDT discussions in current best‐practice recommendations, ultimately improving both MDT efficiency and patient outcomes.

The study has limitations. This was a single centre evaluation performed within a large tertiary referral network and may not be directly generalisable to all renal cancer services. In addition, whilst improvements in workflow efficiency were demonstrated, longer term assessment of patient outcomes, treatment timelines, and clinician experience following pathway implementation remains necessary. Successful implementation also depends on robust triage systems, high‐quality imaging and timely specialist radiological review. Nevertheless, this study provides an early evaluation of a complexity stratified MDT model within renal cancer services.

As renal cancer care continues to evolve alongside increasing service centralisation and treatment complexity, conventional MDT structures will require adaptation to remain sustainable. Focused specialist pathways for protocolised lower complexity cases may allow tertiary MDTs to prioritise patients requiring the highest level of multidisciplinary oncological expertise. We summarise our recommendations based on this evaluation in Table S1.

## Disclosure of Interests

The authors declare no conflicts of interest.

## Supporting information


**Table S1.** Recommendations to optimise MDT meetings.
**Fig. S1.** Management decisions for patients with T1a renal tumours across different age groups.
**Fig. S2.** Management decisions for patients with T1b renal tumours across different age groups.
**Fig. S3.** Management decisions for patients with T3 and T4 renal tumours across different age groups.

## Data Availability

The data that support the findings of this study are available from the corresponding author upon reasonable request.
