# Supplementary material for: Towards sustainable multidisciplinary renal cancer tumour boards: redesigning the referral pathway
Source: BJU Int. 2026 Oct 1;138(6):1133–41. doi: 10.1111/bju.70451 (PMC13741112; doi:10.1111/bju.70451)
Supplement: Supplementary file 1 [file BJU-138-1133-s001.docx]

# Supplementary Figures

## Supplementary Table 1

| **Recommendations for MDTs** | |
| --- | --- |
| **Recommendations** | **Explanation** |
| **Cases Stratified by Complexity** | Allows appropriate allocation of MDT resources |
| **Standardised Referral Criteria** | Clear requirements for referral to minimise unnecessary discussions |
| **Established Protocol-Driven Management** | Allows clear pathway and efficient decision-making |
| **Complete Expert Radiology Review of Renal Lesions Prior to MDT Referral** | Minimises ambiguity of referrals |
| **Prospective MDT Database** | Allows discussions and outcomes to be tracked and monitored |
| **Regular Monitoring of Efficiency** | Periodic audits and evaluations to improve efficiency and outcomes |

Table S1: Recommendations to optimise MDT Meetings

## Supplementary Figure 1

Figure S1: Management decisions for patients with T1a renal tumours across different age groups

## Supplementary Figure 2

Figure S2: Management decisions for patients with T1b renal tumours across different age groups

## Supplementary Figure 3

Figure S3: Management decisions for patients with T3 and T4 renal tumours across different age groups
